# Editorial: Dual role of inflammatory mediators in cancer immunotherapy

**DOI:** 10.3389/fimmu.2023.1229355

**Published:** 2023-07-31

**Authors:** Xiaoli Wang, Jiashuai He, Jinghua Pan

**Affiliations:** ^1^ West General Department, The First Affiliated Hospital of Jinan University, Guangzhou, China; ^2^ Department of General Surgery, the First Affiliated Hospital of Jinan University, Guangzhou, China

**Keywords:** cancer immunotherapy, inflammatory mediators, tumor microenvironment, tumor mutation burden, dual role

Cancer immunotherapy has emerged as a promising treatment strategy for various types of cancers (1). However, the success of immunotherapy largely depends on the ability of the immune system to recognize and eliminate cancer cells. Inflammatory mediators play a crucial role in modulating the immune response to cancer cells. While some inflammatory mediators promote the activation of immune cells and enhance their anti-tumor activity, others can suppress the immune response and promote tumor growth (2). Therefore, understanding the dual role of inflammatory mediators in cancer immunotherapy is crucial for developing effective immunotherapeutic strategies. Our Research Topic aims to provide an overview of the current knowledge on the role of inflammatory mediators in cancer immunotherapy and their potential as therapeutic targets.

Immunotherapy has revolutionized cancer treatment, but many patients remain unresponsive due to the immunosuppressive tumor microenvironment (TME). Tumor-associated macrophages (TAMs) play a crucial role in shaping the TME by interacting with intratumoral T cells. Han et al. provided a thorough overview of how TAMs change their polarization to affect T cells within the tumor, emphasizing their communication with other cells in the TME and their metabolic competition. They summarized that pro-inflammatory factors include Tumor necrosis factor-alpha (TNF-α), Interferon-gamma (IFN-γ), MHC-II, and co-stimulatory molecules such as CD80 (B7-1) and CD86 (B7-2). Therefore, TAMs show potential as an effective means to control inflammation in the TME, enhancing the activation of antitumor immunity while reducing the negative effects of protumor exhaustion and desmoplasia.

The tumor mutation burden (TMB) is also a critical marker that affects the TME in cancer immunotherapy. Wang et al. discuss the limitations of using a one-dimensional numerical representation of non-synonymous genetic alterations, known as tumor mutation burden (TMB), to measure the immunogenicity of tumors. The authors propose that TMB should be segmented into higher dimensional feature vectors to more accurately measure the foreignness of tumors. They developed a model called TMBserval that integrates multiple-instance learning with statistics to create a statistically interpretable model that addresses the broad interdependencies between multidimensional mutation burdens and decision endpoints. The model was tested on data from 137 actual patients and demonstrated the ability to discriminate between patient groups in a high-dimensional feature space, expanding the beneficiary population of immunotherapy.

In this Research Topic, Zhang et al. further discussed the role of adipose tissue in age-related physiological dysfunctions, particularly as a source of chronic inflammation. Aging causes changes in adipose tissue, including fat redistribution, a decline in adipose progenitor and stem cells, accumulation of senescent cells, and dysregulation of immune cells. Inflammaging in adipose tissue contributes to adipocyte hypertrophy, fibrosis, and ultimately, adipose tissue dysfunction, as well as age-related diseases such as diabetes, cardiovascular disease, and cancer. The relationship between cancer and inflammation of adipose tissue is multifaceted. Inflammation in adipose tissue can create an environment that favors the initiation and growth of cancer cells (3). It can induce DNA damage, promote genetic mutations, and disrupt normal cellular processes, leading to the uncontrolled proliferation of cancer cells. Moreover, chronic inflammation can support the survival and spread of cancer cells by promoting angiogenesis (formation of new blood vessels) and facilitating the invasion of nearby tissues (4, 5). One key aspect of cancer-associated inflammation is the presence of a proinflammatory microenvironment. Additionally, obesity has been linked to adipose tissue inflammation, which has emerged as a potential driver of cancer. Adipose tissue, or fat tissue, is not merely an energy storage depot but also an active endocrine organ. In obese individuals, adipose tissue can undergo significant changes and become inflamed. Adipocytes (fat cells) release proinflammatory cytokines, such as tumor necrosis factor-alpha (TNF-α), interleukin-6 (IL-6), and C-C Motif Chemokine Ligand 20 (CCL20) (6), which can promote chronic inflammation. This chronic inflammation in adipose tissue has been associated with an increased risk of developing several types of cancer, including breast, colorectal, and pancreatic cancer, among others. Therefore, adipose tissue inflammation, along with other factors, can significantly contribute to the pathogenesis of cancer. Exploring the complex interplay between inflammation and cancer opens avenues for novel therapeutic interventions and emphasizes the importance of addressing chronic inflammation as a preventive measure against cancer. The article outlines the molecular and signaling pathways involved in adipose tissue inflammaging and proposes potential therapeutic targets to alleviate age- and cancer-related problems.

Further biomarkers and novel immunoregulatory mechanisms were also identified in this Research Topic. Wu et al. found that Aryl hydrocarbon receptor nuclear translocator-like 2 (ARNTL2) is a potential oncogene that plays a role in tumorigenesis and cancer immunity. High levels of ARNTL2 indicate an immunosuppressive tumor environment. Combining ARNTL2 targeting with ICI therapy could benefit cancer patients. Zhang et al. found that CD44 is highly expressed in malignant gliomas, particularly in cases without 1p/19q codeletion, IDH wild-type, and mesenchymal subtypes in GBM samples. Its expression is strongly correlated with stromal and immune cells in the glioma microenvironment. CD44 may be a useful biomarker for predicting immunotherapy responses and mediating the expression of PD-L1. The RUNX1/CD44 axis may promote glioma proliferation and migration, making CD44 a potential target for glioma immunotherapy or a prognostic biomarker. Arra et al. revealed the role of the co-inhibitory receptor PD-1 in modulating the differentiation and effector function of Tc17 cells, which produce interleukin-17 and generally have a suppressed cytotoxic nature. The study found that PD-1 inhibited the expression of IL-17 and Tc17-supporting transcription factors, as well as the expression of the type17-polarizing cytokine IL-21 and the receptor for IL-23. However, PD-1-/- Tc17 cells were highly efficient in rejecting established B16 melanoma *in vivo* and displayed Tc1-like characteristics ex vivo. The absence of PD-1 signaling in Tc17 cells increased the expression of stemness and persistence-associated molecules, indicating plasticity in relation to cytotoxic Tc1 cells driven tumor rejection. The findings suggest that PD-1 plays a central role in suppressing Tc17 differentiation and its plasticity in relation to cytotoxic Tc1 cells driven tumor rejection, making it an efficient therapeutic target for inducing tumor rejection. This mechanism further expands our thinking to develop more related treatment strategies based on PD-1 and PD-L1 (7).

In summary, the Research Topic of *“Dual Role of Inflammatory Mediators in Cancer Immunotherapy”* highlights the dual role of inflammatory mediators in cancer immunotherapy and presents a promising prospect for the development of effective cancer treatments. On one hand, inflammatory mediators can promote tumor growth and progression by suppressing the immune system and promoting angiogenesis. On the other hand, they can also stimulate the immune system and enhance the efficacy of immunotherapy. By understanding the complex interactions between inflammatory mediators and the immune system, researchers can develop novel strategies to harness the immune system’s power to fight cancer. For example, targeting specific inflammatory mediators that suppress the immune system could enhance the efficacy of immunotherapy by removing the barriers that prevent immune cells from attacking cancer cells. Furthermore, the dual role of inflammatory mediators in cancer immunotherapy also highlights the importance of personalized medicine. By analyzing the inflammatory profile of individual patients, clinicians can tailor treatment strategies to optimize the immune response and improve outcomes. Overall, the prospects of the dual role of inflammatory mediators in cancer immunotherapy are promising and offer a new avenue for the development of effective cancer treatments.

## Author contributions

XW drafted the manuscript. JH and JP revised the manuscript. All authors listed have made a substantial, direct, and intellectual contribution to the work, and approved it for publication.
